# Strategies to support midlife women to reduce their alcohol consumption: an Australian study using human-centred design

**DOI:** 10.1093/heapro/daad175

**Published:** 2023-12-21

**Authors:** Mia Miller, Sandra Kuntsche, Emmanuel Kuntsche, Megan Cook, Cassandra J C Wright

**Affiliations:** Menzies School of Health Research, Charles Darwin University, Darwin, Australia; Centre for Alcohol Policy Research, La Trobe University, Melbourne, Australia; Centre for Alcohol Policy Research, La Trobe University, Melbourne, Australia; Centre for Alcohol Policy Research, La Trobe University, Melbourne, Australia; Institute for Social Marketing, University of Stirling, Stirling, Scotland; Menzies School of Health Research, Charles Darwin University, Darwin, Australia; Institute for Social Marketing, University of Stirling, Stirling, Scotland; Burnet Institute, Melbourne, Australia

**Keywords:** alcohol, participatory research, women, human-centred design, midlife

## Abstract

Alcohol consumption is causally associated with long-term health-related consequences, such as cancer and cardiovascular disease, and short-term harms, such as accidents and injuries. Alcohol consumption has increased among midlife women (aged 40–65) over the last two decades in high-income countries. This study aimed to centre women’s voices by using co-design methodologies to investigate what women identify as strategies that could assist them and other women their age to reduce their alcohol consumption. Human-centred design workshops were undertaken with 39 women, and conventional qualitative content analysis was used to analyse information from written workshop materials to develop categories in the data and count their occurrence. Six categories, or strategies, emerged, listed here from most to least represented: ‘Participate in alternative activities to drinking alcohol’, ‘Track alcohol consumption and set goals’, ‘Seek support from family and friends’, ‘Drink alcohol-free beverages’, ‘Reduce supply of alcohol in the home’ and ‘Seek professional support’. Our findings identify strategies that are realistic and feasible to midlife women; our sample, however, likely reflects a more affluent subsection of this group, and as such, any focus on individual-level strategies must be complemented by policies that increase equitable access to healthcare and act on the social and commercial determinants of health. An intersectional approach to alcohol and other drug research is required to examine how the interplay of gender and other markers of social identities shape alcohol consumption.

**Contribution to Health Promotion**

- The alcohol consumption of midlife women is increasing, yet they are historically an understudied group.
- Women’s voices must be centred in the development of health promotion programs and policies. This can be achieved through using human-centred co-design methodologies.
- Women are interested in reducing their alcohol consumption by participating in alternative activities to drinking, tracking their alcohol consumption and setting goals, seeking support from family and friends, drinking alcohol-free beverages, reducing the supply of alcohol in the home and seeking professional support.
- For these strategies to be enacted equitably, the health promotion community must develop targeted resources, advocate for better access to health services and sustain a focus on the social and commercial determinants of health.

## BACKGROUND

Alcohol consumption is a leading risk factor for the global disease burden and there is no safe level of use because of its carcinogenic effects (Rumgay *et al.*, 2021). While there have been declines in overall population alcohol consumption (Smith and Foxcroft, 2009; Grucza *et al.*, 2018; White *et al.*, 2018), recent studies from high-income countries such as Australia, the United Kingdom and the United States have shown significant increases in midlife women’s drinking (midlife is defined for the purpose of this research as 40–65). For example, over an almost 20-year period in Australia (2001–19), long-term risky drinking (2 + standard drinks a day) increased from 8.8% to 11.7%, and risky-single occasion drinking (5 + standard drinks a day) from 13.5% to 19.8% amongst midlife women(Miller *et al.*, 2022).

Historically, limited focus in the literature has been placed on the alcohol consumption of women, and particularly women in midlife (Hunt *et al.*, 2016). Over the last decade, a body of qualitative research has emerged focusing on this group. These studies paint a complex picture of midlife women’s alcohol consumption, demonstrating that it is shaped by the interplay of individual, social, societal and commercial factors (Kersey *et al*., 2022). Additionally, research suggests that the period of midlife is not homogenous, with different biographical transitions occurring for women throughout this period that differentially impact affect, stress and in turn, alcohol consumption, (Lunnay *et al.*, 2023). Additionally, midlife women are not a homogenous group, with differences in socioeconomic status and social standing impacting how women respond to changes in midlife using alcohol (Lunnay *et al.*, 2022a, 2022b; Ward *et al.*, 2022).

While alcohol consumption is a multi-layered behaviour, research has shown consistency in the routines and rituals that midlife women partake in around drinking, with many of these routines enacting gendered identities. For example, 'me time' is described by many midlife women as a routine when they drink alcohol at home at the end of the day, either alone or with a partner, to demarcate the departure from their gendered caring responsibilities and roles in midlife (Emslie *et al*., 2015; Jackson *et al.*, 2018; Kersey *et al*., 2022). While some midlife women describe this drinking as an act of relaxation and ‘self-care’ (Kersey *et al*., 2022; Wright *et al*., 2022), others report that they use alcohol to deal with stress or negative emotions (Emslie *et al*., 2011; Drabble and Trocki, 2014)—and this distinction can be seen along class lines, whereby more affluent women report increased access to resources that means they are not as reliant on alcohol to cope (Ward e*t al*., 2022). For some women, alcohol consumption is also a highly sociable activity, with women reporting drinking for pleasure in social settings, while also acknowledging that they experience social pressure from their peers to drink (Emslie *et al*., 2011, 2017).

As our understanding of midlife women’s drinking behaviours expands, women need to be enabled and empowered to be part of the decision-making around strategies that may be implemented to assist them in reducing their alcohol consumption. Increasingly, human-centred design approaches are being used in public health research as a way to centre the voices of groups of interest and foster understanding and empathy (Romero and Donaldson, 2023). This methodology focuses on finding solutions to complex problems through collaboration between members of the group of interest, designers and subject matter experts through stages of inspiration, ideation and implementation (Davis *et al*., 2020; Romero and Donaldson, 2023). This level of interaction can create feelings of trust and mutuality between participants and researchers (Bazzano *et al*., 2017) which can facilitate engagement on sensitive or stigmatized topics (Davis *et al*., 2020). Beyond traditional focus group or interview methods, human-centred design uses a combination of non-traditional verbal techniques, such as storytelling and role-playing, and written/visual techniques, such as mapping out timelines using icons and drawing representations of ideas (Davis *et al*., 2020). To our knowledge, no studies to date have used co-design processes with midlife women around their alcohol consumption. The aim of this study was to use the principles of human-centred design to support midlife women to identify strategies or solutions they think could assist them and other women their age to reduce their drinking.

## METHODS

### Participants and recruitment

We used convenience sampling to recruit participants through paid Facebook advertisements targeted to women aged 40–65 years residing in Melbourne and Canberra. Individuals who clicked on the advertisement were presented with a brief screening survey. Participants were eligible if they identified as a woman, consumed alcohol at least once a week, and were fluent in English. Participants were ineligible if they had a self-identified current or previous alcohol or other substance use disorder.

### Study design

We conducted workshops with 39 women between June and October 2019, with two to nine participants per workshop. Each workshop was facilitated by a pair of researchers who identify as women, and each pair included one woman in midlife. Workshops were held at flexible times that catered to participants’ schedules, including evening and weekend sessions, and ran for approximately three hours each. Participants were offered a $40 gift voucher in appreciation of their time. Each workshop was audio recorded, and multiple recorders were used when participants were working in groups to ensure all discussions were captured.

The workshop content was designed as part of a larger project to inform the development of a digital alcohol intervention for midlife women. The workshop structure and the included activities were formulated in collaboration between the research team and a design anthropologist from a digital design agency, and mapped across four iterative phases of human-centred design: *understand, define, ideate* and *design* (Davis *et al.*, 2020). To address the specific research aim of this study, the design thinking tool of ‘personas’ was used. Personas are fictitious characters who represent the group of interest, and using them in co-design enables individuals to generate rich data about their needs while avoiding disclosing their own personal information if they are not comfortable doing so (Fuglerud *et al.*, 2020). Participants were asked in groups of two to three to design a persona of a midlife woman by answering several prompts about her demographic characteristics, marital status, worries and stressors, likes and dislikes and current perceptions and use of alcohol. Participants were then prompted to address the question ‘How could you help your persona to change her alcohol consumption?’ and asked to individually write down four possible strategies. Participants were instructed to discuss these solutions in pairs, and then with the larger workshop group and facilitators.

Ethics approval for this study was received from the La Trobe University Human Research Ethics Committee (HEC19041).

### Analysis

Our analysis used a conventional approach to qualitative content analysis (Hsieh and Shannon, 2005). Qualitative content analysis as a whole is a process designed to condense raw data into categories or themes (Cho and Lee, 2014), with a conventional approach differing from other qualitative content analysis approaches in that categories are derived inductively from the data, rather than based on pre-existing theory (Hsieh and Shannon, 2005). This approach fits well with our research question and aims, which are to centre women’s voices by reporting on strategies they devise themselves that can be translated into practical outcomes or solutions.

The units of analysis for developing the categories were the four written responses from each participant. These were entered verbatim into an Excel spreadsheet. All of the responses were read multiple times, and responses that provided similar answers were grouped together into categories, with each category then given a name. Some of the responses were allocated to two categories, for example: ‘Set up a regular time to socialise with friends at the park every Tuesday evening instead of at the pub and bring a different non-alcoholic beverage each time’ sat within two categories: ‘Participate in alternate activities to drinking alcohol’ and ‘Drink alcohol-free beverages’. We then counted the occurrence of each category to see how commonly it was mentioned in the data. When writing about each category, we reviewed the transcripts from the workshops to understand how women spoke about their strategies and what types of agreement or divergence arose in group discussion. Quotes in this paper are drawn from both the written responses and the transcripts.

## RESULTS

Table 1 shows the demographic characteristics of the sample. The average age of participants was 59, and participants were predominantly employed full-time or retired. Most participants had a university degree, were born in Australia and had children, and half lived with a partner and/or child. An emerging research base has been taking a classed approach to alcohol consumption, exploring the ways in which women’s relationship with, and willingness and ability to, reduce alcohol consumption is tied in with their social class (Lunnay *et al*., 2022a, 2022b; Ward *et al.,* 2022). Our study was not designed within this paradigm, and as such we cannot be explicit about the social class of our participants; however, based on the demographic data we collected, we can classify them as likely belonging to a more affluent subsection of society. We will interpret the implications of our findings in the discussion in relation to our sample.

**Table 1:** : Participant demographics

	*n* ^a^ (%)
**Mean age (IQR)**	59 (8)
**Employment**	
Full-time	13 (37)
Part-time	8 (21)
Self-employed	3 (8)
Retired	13 (37)
**Highest level of education**	
Tertiary	35 (95)
Higher school or less	2 (5)
**Country of birth**	
Australia	33 (89)
Other	4 (11)
**Household competition**	
Currently living with partner	25 (58)
Currently live with children	19 (51)
**Have children**	
Yes	33 (89)
No	4 (11)
**Family composition**	
Number of children (median, range)	2 (1–4)
Age of children	28 (9–46)

^a^Total *n* = 37, data missing for two participants.

Table 2 shows the categories and how often they were identified in the data.

**Table 2:** Categories identified in the data

Category	Count
Participate in alternative activities to drinking alcohol	35
Track alcohol consumptions and set goals	23
Seek support from family and friends	16
Drink alcohol-free beverages	15
Reduce supply of alcohol in the home	8
Seek professional support	7

While participants originally used the third person when describing their persona and writing their original written responses, when discussing their responses with other participants and the facilitator many shifted into discussing ideas linked to themselves or other women their age. As such, and to aid comprehension, we will not refer to personas in the results, and rather discuss the findings as what women were suggesting for themselves, and other women like them.

### Participate in alternative activities to drinking alcohol

Engaging in physical activity or new hobbies was a strategy proposed by many women in our sample. A plethora of alternative activities were suggested, including walking, exercise classes, creative hobbies and meditation. While this was a common strategy consistently identified in the workshops, the underlying reasons for engaging in this strategy varied for different women. For some women, activities to replace the rituals around using alcohol to cope with stress or unwind were suggested, as expressed by Sheila in *Workshop 5: ‘relaxation, yoga, meditation to cope with her life instead of using alcohol’.* These women believed that certain hobbies could provide an appealing alternative to reduce the negative emotions that were common triggers for their alcohol consumption.

For other women, they envisioned that new hobbies would change their routine, providing an alternative focus, or simply resulting in them having less time available to drink alcohol, as Helen from *Workshop 7* identified: *‘She can have a complete change of routine, like exercising when it would normally be “wine time.”’* Bettina from *Workshop 6* expressed interest in walking or exercising before meals as a way to *‘reduce the time spent drinking before dinner’.*

Women we spoke to also suggested breaking the routine of social drinking with peers by engaging in alternate activities such as a joint gym or art class, as this would mean a woman could: *‘put herself in social situations where the focus isn’t on alcohol’* (*Dianne, Workshop 5)*. They felt that this approach would remove the pressure to socialize in alcohol-filled environments, such as restaurants or bars, which has been identified as a common practice among midlife women (Wright *et al.*, 2022).

### Track alcohol consumption and set goals

In actioning reduced alcohol consumption, many women within our sample identified that tracking their alcohol consumption, setting goals, and rewarding themselves for achieving these, could be helpful approaches. The type of goals women in our study suggested varied greatly, ranging from only drinking in the company of others, only drinking with food, having a certain number of alcohol-free days every week, limiting the number of drinks consumed on any one day, and participating in a month of abstinence such as Dry July.

Women in our workshops suggested that a phone application or keeping a physical drinks diary or chart may assist with personal monitoring, though they did not make explicit suggestions about existing options. It was also suggested that tracking other things alongside alcohol consumption, like sleep, emotions, and financial savings, could enable a woman to observe other changes in her life as her alcohol consumption reduces. Very few of the identified rewards were related to improved health or well-being in the longer term, although one or two women earlier in the workshops had identified that they were interested in cutting down on alcohol to assist with weight loss. This focus on tracking tangible, more immediate benefits aligns with findings from previous studies where midlife women appear to be concerned with the disruptions alcohol can cause in their daily lives (Lyons *et al.*, 2014; Muhlack *et al.*, 2018; Dare *et al*., 2020) rather than with long-term health risks. Similarly, the rewards women focused on were around the shorter-term gains from reducing drinking, particularly from a financial perspective, such as using the money saved to buy themselves a gift, have an evening out or go away for a weekend, as Erica *[Workshop 4]* said: *‘She could decide on a series of rewards for time periods when she has kept to her goal, e.g., spend $100 on something she wants after a month of not drinking on weekdays’.*

### Seek support from family and friends

Women commonly suggested that they could inform friends and family of their intentions to change their alcohol consumption, identifying that this would either help keep them accountable to their commitments to reduce their alcohol consumption or it would reduce the possibility of pressure from others to drink. In *Workshop 6,* Bettina identified the strength that a woman’s immediate support network could provide in achieving personal goals: *‘Women do tend to influence one another and support one another. And that’s where that influence will come from, from that network’.* This finding suggests that an alternative to drinking non-alcoholic drinks in social settings in order to fit in could be for women to engage in conversations around alcohol with their peers to reduce the social pressure at the source.

Another commonly proposed strategy was that women could reduce their alcohol consumption in partnership with friends or family so that their health could be a joint focus. Jenny *(Workshop 9*) suggested that a woman could: *‘Enlist her partner’s support—get him to cut down on his drinking too because it is also better for his health’.* This is in line with suggestions from past research, which has highlighted the importance of seeing women’s alcohol consumption in the context of wider familial and non-familial relationships (Jackson *et al*., 2018), and therefore of working with women to enable their networks to support their desired change.

### Drink alcohol-free beverages

Another strategy suggested by women in our sample was to replace the beverages they consumed with alcohol-free options. This was a strategy that was identified as useful in both social settings, and in relation to home drinking practices. Women who endorsed this idea suggested that they could consume mocktails or other alcohol-free drinks when socializing at licensed premises, as this would allow them to fit in while masking their non-drinking. For Jacky, drinking alcohol-free alternatives could allow a woman to continue to socialize with her peers without needing to consume alcohol: *‘Maybe talk about some non-alcoholic drinks that she can have, there are things you can do to make it look like you’re having a drink when you’re not having a drink’ (Workshop 8).* As has been identified in other studies (Lyons *et al.,* 2023), some women in our sample described drinking alcohol at home on weeknights after a busy day as a ritual that is entangled with the gendered caring and housework roles they engage in. For these women, they were interested in substituting their alcohol consumption in these situations for a non-alcoholic beverage. For women who explicitly identified that they drank due to the enjoyment of the taste of alcohol, particularly wine, women suggested the consumption of teas, sparkling water and smoothies as substitutes that would still bring enjoyment: *‘[I could] increase [my] water intake and make a lovely chai at home to enjoy the taste of it’ (Helen, Workshop 7).* Janine from *Workshop 1* suggested using the money she would usually spend on wine to buy an expensive non-alcoholic drink that feels like a ‘treat’, and thus still acts as a reward at the end of a busy day: *‘You come home and you’re like “I deserve this, and I’m going to have this.”’*

### Reduce supply of alcohol in the home

Women from our sample identified that having alcohol readily available in their homes was one way that routines and rituals around drinking were perpetuated. Therefore, it was proposed that another way to disrupt their drinking habits was to buy and store less alcohol in their homes, with Bettina from *Workshop 5* suggesting women could *‘Stop ordering wine, because if it’s not there, you can’t drink it’.* Some women in our sample identified that they were primarily wine drinkers who ordered wine in bulk or bought it from local wineries. While women described attending wineries as a pleasurable activity, and ascribed pleasure to enjoying the taste of drinking wine, they also identified that having large supplies of alcohol in their homes was a barrier to change. Isabel *(Workshop 4)* felt that more thoughtful and purposeful purchasing of alcohol could disrupt the automated routines of using alcohol at home, thereby reducing the frequency of consumption: *‘Rather than having a magnificent cellar, have a small stock of something they are going to drink together’.* These findings also highlight the tensions between enjoyment and restraint evident within the act of consuming (and also in this case buying) alcohol, and point to the often-overlooked concept of pleasure as one that needs to be considered for midlife women wanting to reduce their alcohol consumption. For women who regularly socialized at wineries, the notion of engaging in alternative, alcohol-free activities with their friends was raised again here, with Shelley from *Workshop 5* stating that during Dry July, her group of friends and partners *‘played cards, backgammon, lawn bowls… went to the movies a few times’* as a way to maintain pleasure outside of alcogenic environments, which would then reduce their opportunities to purchase alcohol to store in their homes.

### Seek support from healthcare professionals

Participants also suggested that women could seek help and information about their alcohol consumption outside the family and friendship group. They suggested visiting a general practitioner (GP), a therapist, a specialized alcohol or other drug counsellor or to call a helpline. There was some debate within the workshops about which speciality was best to support women in changing their alcohol consumption. Some participants suggested women could speak to their regular GP, describing them as an important point of contact due to their knowledge of a woman’s history and specific risk factors. However, another participant, Janine *[Workshop 1]* flagged her perception of the difficulties in seeking GP support and suggested seeing a psychologist or counsellor as these specialities *‘certainly have more time – GPs have about 10 minutes!’* Another barrier raised to accessing GP support was that some women might downplay their alcohol consumption when talking to a GP due to shame or fear of stigma, as Carmen from *Workshop 5* stated: ‘*There’s this sort of shame thing as you might get judged if you have 6 glasses of wine at night’.* One woman, Sarah from *Workshop 5*, suggested that developing a specific women’s health check including a component on alcohol consumption, with resources tailored for midlife women, could be a way to overcome the aforementioned barriers, proposing that *‘maybe it needs to come as part of a GP visit, women go to have a pap smear or whatever and maybe that needs to be an extra step’.* Another woman in this workshop, Daphne, agreed with this suggestion, emphasizing that this approach could *‘make it more of a health than a social problem, so it’s less stigmatised’.* Within the workshops, there also appeared to be a lack of knowledge amongst participants as to what level or patterns of drinking may require support from different types of practitioners, suggesting that a GP as a first contact point could help guide women to appropriate support.

## DISCUSSION

The aim of our study was to use the principles of human-centred design to enable midlife women to identify strategies that they can employ to reduce their alcohol consumption. We used the activity of ‘personas’ to facilitate this dialogue, with women discussing ideas that they believed would be helpful for their persona, themselves, and other women like them. Strategies suggested included picking up new hobbies or activities, tracking their alcohol consumption and setting goals, seeking support from family and friends, trying alcohol-free alternatives, avoiding having alcohol in the home, and seeking professional support. When talking about these strategies, women also reflected back how and why these particular approaches might work for them, and many of these observations align with the findings of previous qualitative studies on midlife women’s drinking, such as that many women drink to cope (Kersey *et al.*, 2022) and that alcohol is an ordinary commodity embedded and easily accessible in their homes (Lyons *et al*., 2023). As such, while the strategies identified in our study are primarily operating at an individual level, they tap into aspects that shape the social practices and social norms of drinking among midlife women (Lunnay *et al*., 2022a, 2022b).

Our findings can help inform the design of policies and programs such as brief interventions and training for health care professionals, as well as provide direction on future research efforts. Actioning disruptions to rituals seems to be a feasible and acceptable way for women to reduce their alcohol consumption, but may not be as easy as simply encouraging women to try new activities, as the routines which women want to replace are heavily ingrained in their lives (Wright *et al*., 2022; Lyons *et al*., 2023). As such, tailored health promotion resources and digital interventions that focus on providing women with the materials and competencies to form new social practices around non-drinking are needed. To date, few alcohol-focused interventions have been designed by and for women, and further efforts are needed to develop and test digital interventions. Some of our findings could shape the content of such interventions. For example, most brief interventions take an individualistic approach (McCambridge, 2021), but our results highlight that including a woman’s support network in such interventions would be beneficial. Additionally, women showed an interest in goal setting and tracking their alcohol consumption and were motivated by implementing goals that felt realistic and rewarding for themselves. As midlife women tend to focus on the short-term harms of alcohol consumption (Dare *et al.,* 2020), personalized goal setting could be a useful component of interventions, with research showing that individuals display greater commitment to and self-efficacy relating to goals when they are participatively set rather than assigned (Lozano and Stephens, 2010).

Women identified health practitioners as a potential source of support, with a focus of discussion on GPs. This is in line with findings from a previous study which demonstrates that women, particularly those in middle and upper classes, view health practitioners as a trustworthy source of health information (Meyer *et al*., 2022). There are large discrepancies in whether GPs address alcohol consumption in appointments that depend on their confidence in managing alcohol issues, the short length of appointments, their perceptions on how discussing alcohol may influence the doctor–patient dynamic, and a range of other factors (Tam *et al*., 2013; Miller *et al.*, 2016). Training GPs on the importance of engaging with midlife women on alcohol is needed, particularly if women, as reported in our study, feel shame or stigma about drinking, and thus may not raise this issue independently. In particular, educating GPs on how to engage with midlife women on alcohol issues and consequences that can exist across the continuum of perceived ‘light to heavy’ drinking (Morris *et al.*, 2023), and the types of tailored information or referrals required, is needed. The financial barriers to accessing GPs likely acts as a barrier for less affluent women to access these services, and expanding access to bulk billing (fully subsidized) services under Medicare, Australia’s publicly funded health insurance scheme, is needed.

Another strategy that women suggested to disrupt the rituals around alcohol consumption was to drink non-alcoholic alternatives, as this would allow for the masking of non-drinking in social settings or act as a stand-in beverage from a taste perspective in the home. From a research perspective, most studies of No and Low alcohol drinks (NoLos) have been market research and/or alcohol industry-funded, or conducted by researchers with alcohol industry ties. NoLos may act as an effective harm-reduction tool if individuals substitute some of their regular alcohol consumption with these products (Miller *et al.,* 2022). Research is needed that takes a public health harm reduction perspective to dissect the ways in which midlife women specifically perceive that NoLos may or may not replace the experiences of drinking alcohol. Such research should also consider drinking motives and social class, as NoLos may be less effective for women who drink to experience the psychoactive effects of alcohol to help cope with stress or loneliness, which is commonly reported by more working-class women (Lunnay *et al*., 2022a).

The majority of our sample were born in Australia, were tertiary educated and in some form of employment or retired and as such, they likely characterize a more affluent subsection of the population of midlife women. This is important to consider in the context of research which suggests that midlife women’s descriptions of their relationship with alcohol consumption, their reasons for reducing consumption, as well as their ability and willingness to reduce consumption, differ by social class (Lunnay *et al*., 2022a, 2022b), with middle- and upper-class women privileged with having more agency and more tools to deal with stressful life experiences. As such, some of the strategies mentioned by women in our sample may not be realistic or available to other subsets of midlife women due to financial, social, and other restraints. Additionally, while these strategies are approaches that fit well into the way that women in our sample live and talk about wanting to be helped, it is important to consider how women’s imaginations of what might be possible may be limited by what they see around them and the framings that they approach alcohol with. For example, the strategies that women identified were primarily ones they have personal control over, and they did not talk about policies such as outlet density or alcohol advertising as things that needed to change for them to reduce their consumption. Participants’ responses may have been shaped by the way that the study research question was framed to participants, but could equally be due to the neoliberal discourses around alcohol perpetuated by the alcohol industry, and sometimes also by governments and policymakers (Room, 2011; Lencucha and Thow, 2019). Drawing women’s attention to the commercial factors that shape choices and behaviours may be one way to expand the framings of alcohol that women are exposed to (McCambridge, 2021), and human-centred design research could be used with midlife women to design and test messages on the commercial determinants of health. Additionally, policies that address the increasing availability of alcohol (Babor *et al*., 2022), and the pervasive alcohol advertising that targets women (McCarthy *et al*., 2023) and reinforces social norms around drinking (Atkinson *et al*., 2022), are needed. Finally, the social determinants of health must be addressed in order for women to be able to reduce their alcohol consumption. In particular, enabling women to reduce their alcohol consumption requires the consideration and implementation of policies that address gender equity, employment, education and childcare, for instance, as an acknowledgement that the pressures on midlife women to manage multiple competing priorities including work and caring roles impacts their agency over their alcohol consumption (Lunnay *et al.*, 2023). Despite the imperative to acknowledge the central role that environments and systems play in shaping alcohol consumption, approaches implemented at the individual level that are within the realm of reality, particularly for middle and upper-class women with access to resources and agency, may empower women to make change in ways that strategies only focusing on the structural determinants may not.

## CONCLUSIONS

Overall, our study provides insight into strategies that midlife women suggest may be suitable for women their age to reduce their alcohol consumption. These strategies included disrupting the rituals around alcohol consumption by engaging in new hobbies and activities, drinking alcohol-free alternatives, and reducing the supply of alcohol in the home. They also incorporated setting goals and tracking alcohol consumption, and seeking support from friends and family, and from medical practitioners. Designing tailored health promotion resources and brief interventions for midlife women that help facilitate the adoption of new reduced or non-drinking social practices is needed, as are resources that can support women within their social networks to discuss and navigate social pressures to drink. Improving access to GPs and psychological support is also required through reforms to the healthcare system, as well as ensuring that GPs are trained and confident to support women who seek help. Continuing to advocate for better regulation of alcohol advertising is fundamental to shift the perceptions that alcohol consumption is integral for socialization. Our sample may reflect a more affluent subsection of midlife women, and as such a strong focus on the social determinants of health is required. The alcohol and other drug research field has been a late adopter of intersectionality, and our research must examine the ways in which the interplay of gender and other markers of social identities shape alcohol consumption (Hunt and Antin, 2020).
